# Refining Stroke Risk Assessment in Patients with Device-Detected Atrial Fibrillation

**DOI:** 10.3390/jcm14010082

**Published:** 2024-12-27

**Authors:** Andreas Sjøholm-Christensen, Nedim Tojaga, Axel Brandes

**Affiliations:** 1 Department of Cardiology, Esbjerg Hospital—University Hospital of Southern Denmark, Finsensgade 35, DK-6700 Esbjerg, Denmark; andreas.sjoholm-christensen@rsyd.dk (A.S.-C.); nedim.tojaga@rsyd.dk (N.T.); 2 Department of Regional Health Research, University of Southern Denmark, Finsensgade 35, DK-6700 Esbjerg, Denmark

**Keywords:** device-detected atrial fibrillation, subclinical atrial fibrillation, atrial high-rate episodes, atrial fibrillation, oral anticoagulation, direct oral anticoagulation, thromboembolic risk, ischaemic stroke, systemic embolism

## Abstract

Clinical atrial fibrillation (AF) is a well-established major risk factor for stroke and systemic embolism. Pivotal trials have shown that treatment with oral anticoagulation reduces the risk of stroke and systemic embolism in clinical AF with a simultaneous increase in the risk of major bleeding. To help balance the risk of stroke and bleeding in clinical AF, different prediction models including biomarkers and clinical features have been validated. Device-detected AF (DDAF) is also associated with an increased risk of stroke and systemic embolism, but not to the same extent as clinical AF. Two large randomised studies have found significant stroke risk reduction with direct oral anticoagulation in DDAF patients, yet also a significantly increased risk of major bleeding. To date, the question remains how to balance the thromboembolic risk reduction with oral anticoagulation and the increased risk of bleeding in patients with DDAF and to identify the right patients who may benefit from oral anticoagulant treatment.

## 1. Definition and Relevance of Device-Detected Atrial Fibrillation

Device-detected atrial fibrillation (DDAF) is atrial fibrillation (AF), which is often short-lasting, without obvious symptoms, and only recorded by a cardiac implantable electronic device (CIED), i.e., either a pacemaker or an implantable cardioverter–defibrillator (ICD) with an atrial electrode or an implantable cardiac monitor (ICM). DDAF was initially described in studies of pacemaker and ICD patients and called atrial high-rate episodes (AHREs). Later, the term subclinical AF (SCAF) was used, when an association between SCAF and ischaemic stroke/systemic embolism became evident [1,2,3,4,5]. Studies show that up to one-third of patients over the age of 65 years with either pacemakers or ICMs have DDAF [4,6,7]. A meta-analysis of cohort studies confirmed a DDAF prevalence of 28.1% and found it is associated with increasing age, hypertension, heart failure, and prior stroke or transient ischaemic attack (TIA). However, substantial heterogeneity between studies was identified [8], which reflects the heterogeneity of the CIED population, thus limiting the generalisability of the findings from single studies to the whole population susceptible to DDAF.

Bradyarrhythmias requiring CIED implantation often occur with increasing age [9,10]. Moreover, the general life expectancy has been steadily increasing up until 2019, as reported by the World Health Organisation (WHO) [11], thus extending the population in need of CIED. The latest worldwide survey on pacemaker and ICD implantations, from 2009, included 61 countries and found 737,840 new pacemakers and 222,407 new ICD implantations, which was an overall increase in the number of implantations since 2005 [12]. More recent data from the National Danish Pacemaker and ICD Register show an almost 2-fold increase in new pacemaker implantations and a 2.5-fold increase in new ICD implantations per year from 2003 to 2022 [13].

Facing such a large group of patients at risk of developing DDAF, many questions arise regarding appropriate treatment, including the use of oral anticoagulation (OAC).

## 2. Stroke Risk in Patients with DDAF

Clinical AF captured by a 12-lead surface electrocardiogram (ECG) or by ambulatory ECG monitoring with an episode duration of at least 30 s is a well-established major risk factor for stroke with an up to 5-fold increased risk [14,15,16,17]. Likewise, DDAF is associated with an up to 2.5-fold increased risk of stroke or systemic embolism, which is approximately half of the thromboembolic risk of clinical AF [4,5,18]. Over the past two decades, several studies have been performed to better understand the thromboembolic risk and how to manage it in DDAF patients.

The ASSERT (Asymptomatic atrial fibrillation and Stroke Evaluation in pacemaker patients and the atrial fibrillation Reduction atrial pacing) trial found a 1.69% annual rate of stroke or systemic embolism among DDAF patients, and this was more than twice as high (3.78%) in those patients who had a CHADS_2_ score > 2 [4]. The LOOP (implantable loop recorder detection of atrial fibrillation to prevent stroke) study compared screening for AF with an ICM with standard care to detect AF and subsequent initiation of OAC in both groups, if AF was detected, in a population at high risk of thromboembolic events. Ninety-one percent of the patients with DDAF-initiated OAC. In total, 315 strokes were recorded and 4.5% of the patients with DDAF had a stroke or systemic embolism corresponding to 0.88 events per 100 person-years [7]. A post-hoc analysis of the LOOP study found no significant difference in severe strokes, defined by the modified Rankin scale, between the ICM and standard care groups (hazard ratio (HR) 0.69 [confidence intervals (CI) 0.44–1.09]). The majority of strokes were ischaemic in both groups, with 87% in the ICM group and 83% in the control group. Study participants who had been diagnosed with AF before the stroke had a significantly higher modified Rankin scale compared with patients without AF, of 3 and 2 (*p*-value < 0.001), respectively [19].

The ARTESiA (Apixaban for the Reduction of Thromboembolism in Patients With Device-Detected Subclinical Atrial Fibrillation) trial (Table 1) was a large double-blind, double-dummy randomised placebo-controlled study that set out to investigate whether treatment with apixaban reduced the risk of stroke and systemic embolism in patients with DDAF compared with aspirin. Study participants had a mean CHA_2_DS_2_-VASc score of 3.9 in both groups. If DDAF duration > 24 h or clinical AF was detected during follow-up, patients were censored and open-label apixaban was initiated. In the intention-to-treat population, stroke or systemic embolism occurred in 55 patients in the apixaban group and in 86 patients in the aspirin group, respectively. Furthermore, there were two systemic embolisms in the aspirin group and none in the apixaban group. In the intention-to-treat analysis, the rate of stroke or systemic embolism per patient-year was low in both the aspirin group and the apixaban group (1.24% and 0.78%, respectively, Table 2), compared with earlier observational studies of DDAF and the aspirin arms of randomised trials of patients with clinical AF [20,21], despite the high nominal risk score [22]. A previous meta-analysis of studies in patients with clinical AF found a minor stroke risk reduction with antiplatelet therapy and one might speculate that treatment with aspirin in ARTESiA may have contributed to the low event rate [23]. According to a subgroup analysis of the ARTESiA trial, 39.4% of patients had a CHA_2_DS_2_-VASc score < 4, 33.6% of patients had a score of 4, and 27% had a score > 4. In the latter group, the rate of stroke and/or systemic embolisms was significantly higher with a rate of 2.25% per patient-year compared with 0.97% per patient-year in the group with a CHA_2_DS_2_-VASc score < 4, both treated with aspirin [24].

The NOAH-AFNET 6 (Non-vitamin K antagonist Oral Anticoagulants in patients with Atrial High-rate Episodes) trial (Table 1) is another large double-blind randomised placebo-controlled study comparing edoxaban with placebo in DDAF patients. If indicated due to other reasons at baseline, treatment with aspirin in the control group was continued. If clinical AF was detected during follow-up, patients were censored and entered open-label edoxaban treatment. As in the ARTESiA trial, the study participants had a high risk of stroke or systemic embolism with a median CHA_2_DS_2_-VASc score of four in both groups. Twenty-two strokes occurred in the edoxaban group and twenty-seven in the control group, corresponding to a stroke rate of 0.9% and 1.1% per patient-year, respectively (Table 2). A composite of stroke and systemic embolism occurred in 25 patients (1% per patient-year) and 38 patients (1.5% per patient-year) in the edoxaban and control group, respectively. Fifty-four percent of patients in the control group were treated with aspirin, which might have contributed to the low thromboembolic rate in the placebo group [25].

A subgroup analysis of pooled data from the ARTESiA and NOAH-AFNET 6 trials found that approximately half of the patients had vascular disease (Table 1), defined as unstable angina, prior myocardial infarction, prior coronary artery bypass grafting or percutaneous coronary intervention, prior stroke/TIA, or established arterial disease. The rates of stroke or systemic embolism in the control groups were higher in patients with underlying vascular disease compared with patients with no vascular disease in both ARTESiA and NOAH-AFNET 6 (1.8% vs. 0.8% and 2.2% vs. 0.8% per patient-year, respectively) [26].

**Table 2:** Event rates per patient-year of selected outcomes in randomised DDAF trials.

	NOAH-AFNET 6 ^a^ [27]			ARTESiA ^a^ [22]		
	Edoxaban 60 (30) mg Daily ^b^	Placebo ^c^	NNT/NNH *	Apixaban 5 (2.5) mg Twice Daily ^b^	Aspirin 81 mg Daily	NNT/NNH *
Stroke or systemic embolism	1.0	1.5	200	0.78	1.24	217
Ischaemic stroke	0.9	1.1		0.64	1.02	
Major bleeding	2.1	1.0	91	1.53	1.12	244
Intracranial haemorrhage	Not reported	Not reported		0.24	0.33	
Cardiovascular death	2.0	2.2		1.47	1.53	
All-cause death	4.3	3.7		5.06	4.82	
Ischaemic stroke	0.9	1.1		0.64	1.02	

^a^ All data reported are intention-to-treat. ^b^ The doses of apixaban and edoxaban were adjusted based on predefined criteria. ^c^ Fifty-four percent were treated with aspirin 100 mg once daily. * NNT number needed to treat per year reported to avoid one ischaemic stroke or systemic embolism, NNH number needed to harm per year reported to cause one major bleeding.

## 3. Association Between DDAF Burden and Thromboembolic Risk

In clinical AF, a greater AF burden is thought to be associated with a higher thromboembolic risk. This is supported by a post-hoc analysis including data from the AVERROES (Apixaban Versus Acetylsalicylic Acid to Prevent Stroke in Atrial Fibrillation Who Have Failed or Are Unsuitable for Vitamin K Antagonist Treatment) and ACTIVE-A (Atrial Fibrillation Clopidogrel Trial with Irbesartan for Prevention of Vascular Events) trials in which patients treated with aspirin had an increasing annual risk of stroke according to their AF phenotype (paroxysmal AF 2.1% risk, persistent AF 3.0% risk, and permanent AF 4.2% risk). In both the paroxysmal and persistent AF groups, the CHA_2_DS_2_-VASc score was 3.1. The permanent AF group had a significantly higher CHA_2_DS_2_-VASc score of 3.6 [28].

The frequency, duration of each DDAF episode, and total burden of DDAF can be relatively easily assessed through constant rhythm monitoring by the CIED. The majority of studies to date have primarily investigated whether the longest duration of DDAF is associated with an increased thromboembolic risk, and the results are conflicting.

In the ASSERT trial, the thromboembolic risk increased the longer the duration of DDAF. In the primary trial report, the duration was split into three subgroups: >6 min–6 h, >6 h–24 h, and >24 h. Each subgroup had a significantly higher risk of stroke or systemic embolism compared with study participants with no DDAF. The highest thromboembolic risk was found in the subgroup with DDAF duration > 24 h (HR 4.96 [CI 2.39–10.3]) [4]. A post-hoc analysis of the ASSERT trial concluded that 11% of the patients had DDAF durations > 24 h, and, compared with patients who had no episodes of DDAF, this was the only group with an increased thromboembolic risk. This subgroup analysis of the ASSERT trial was not powered to detect differences in stroke risk between groups yet provided a signal that longer episodes of DDAF resulted in a higher thromboembolic risk [29].

In the NOAH-AFNET 6 trial, the median duration of the DDAF episodes was relatively short, i.e., 2.8 h (interquartile range 0.8 to 9.4 h), and evenly distributed in both the placebo and edoxaban group. In the ARTESiA trial, only 7.1% of patients had a duration of DDAF between 12 and 24 h six months prior to inclusion. Patients with durations > 24 h were excluded. The majority (35.5%) of patients had DDAF durations of 1–6 h with a mean duration of 1.5 h. A post-hoc analysis of the ARTESiA trial showed no association between the frequency or the duration of DDAF episodes and the thromboembolic risk [30].

In the NOAH-AFNET 6 study patients with DDAF duration > 24 h were included, and a post-hoc analysis showed that the subgroup with DDAF duration > 24 h had a similarly low annual event rate of stroke compared with a DDAF duration <24 h in the placebo group, 0.97% and 0.96%, respectively. In the >24 h group only two ischaemic strokes were recorded. A new composite outcome of ischaemic stroke and systemic embolism excluding pulmonary embolism and myocardial infarction in the post-hoc analysis found a higher annual event rate of ischaemic stroke in the group >24 h compared with <24 h, 6.4% and 2.8%, respectively. Twenty-nine percent in the >4 h duration group compared with seventeen percent in the <24 h duration group developed clinical AF, and as such initiated open-label treatment with edoxaban [31]. The higher amount of open-label treatment with edoxaban in the >24 h duration group might have resulted in a lower annual rate of stroke or systemic embolism.

## 4. Risk of Bleeding

The risk of stroke or systemic embolism in clinical AF is reduced with OAC treatment; however, it increases the risk of bleeding [23]. Direct oral anticoagulants (DOACs) were non-inferior at reducing thromboembolic risk and had a lower risk of intracranial haemorrhage/fatal bleeding compared with warfarin in the pivotal trials. In contrast, they showed a higher risk of gastrointestinal (GI) bleeding, including major bleeding events, when treated with DOACs compared with warfarin [32,33,34,35], except for apixaban which demonstrated a non-significant trend towards fewer GI bleedings [36].

Studies investigating DDAF often have major bleeding as their safety endpoint, and thus more data regarding bleeding risk are emerging in this patient population. The LOOP study had 65 (4.3%) major bleedings in the ICM group and 156 (3.5%) major bleedings in the control group. This corresponded with a non-significantly increased risk of major bleeding in the ICM vs. the control group (HR 1.26 [CI 0.95–1.69]). The trend can be due to more patients (29.7%) being diagnosed with AF in the ICM group compared with the standard care group (13.1%) leading to the initiation of OAC treatment [7].

In the NOAH-AFNET 6 trial (Table 2) major bleeding occurred in 53 patients (2.1% per patient-year) in the edoxaban group and 25 patients (1.0% per patient-year) in the control group (HR 2.10 [CI 1.30–3.38]). The absolute risk increase (ARI) in the edoxaban group was 1.1% per year with a number needed to harm (NNH) of 91 patients being treated to cause one major bleeding. No differences in all-cause mortality or fatal bleeding were detected. More than 50% of patients in the placebo group were treated with aspirin, which may underestimate the increased risk of major bleeding due to edoxaban treatment [25]. The observed incidence of major bleeding in the NOAH-AFNET 6 trial is consistent with data from the ENGAGE AF-TMI 48 trial and clinical practice [27,37].

In the ARTESiA trial, the control group was treated with aspirin, which likely increased the number of major bleeding events as opposed to a control group who did not receive anti-thrombotic drugs. In the aspirin group, 78 major bleeding events (1.12%/patient-year) occurred in contrast to 106 major bleeding events (1.53%/patient-year) in the apixaban group (Table 2), resulting in a significantly higher risk of bleeding in the apixaban group (HR 1.36 [CI 1.01–1.82]). ARI in the apixaban group was 0.41% per year with a NNH of 244 patients being treated to cause one major bleeding. The need for blood transfusion was numerically higher in the apixaban than in the aspirin group (0.89% vs. 0.35% per patient-year). No significant differences were found in all-cause mortality, intracranial haemorrhage, or fatal bleeding between the two groups [22]. A subsequent subgroup analysis divided the patients into three groups, according to their CHA_2_DS_2_-VASc score (<4, 4, or >4) and found an annual rate of major bleeding in the apixaban group of 1.44%, 1.18%, and 2.13%, respectively. The risk of major bleeding when comparing apixaban with aspirin was lower in the group with a score < 4 (HR 1.27 [CI 0.8–2.03]) and slightly higher among those with a score > 4 (HR 1.48 (CI 0.89–2.29) [24].

A meta-analysis including ARTESiA and NOAH-AFNET 6 confirmed that DOACs significantly increased the risk of major bleeding, with 4.8% compared with 3.2% in the aspirin/placebo group (HR 1.62 [CI 1.05–2.05]). DOAC treatment led to ARI of 1.6% with an NNH of 63 patients being treated to cause one major bleeding. No significant differences were found in terms of fatal bleeding and all-cause mortality [30].

A subgroup analysis from pooled data from the ARTESiA and NOAH-AFNET 6 trials found a higher incidence rate ratio (IRR) of major bleeding in patients without vascular disease compared with patients with known underlying vascular disease (IRR 1.93 (CI 0.72–5.20) vs. IRR 1.55 (CI 1.10–2.20)). In both studies, DOAC treatment increased the risk of major bleeding regardless of vascular disease status. The most profound risk increase of major bleeding was found in the NOAH-AFNET 6 data without vascular disease (HR 3.47 (CI 1.49–8.06), probably due to no aspirin treatment in the control group. A very low rate (0.1% per patient-year) of life-threatening bleeding occurred among DOAC-treated patients with underlying vascular disease [26].

## 5. OAC and Thromboembolic Risk Reduction in DDAF

In the LOOP study, OAC was recommended if the duration of DDAF was > 6 min. The treating physician chose which OAC to initiate. In the ICM group with DDAF, 91% initiated OAC treatment compared with 86.5% in the control group with clinical AF. The number of ischaemic strokes in the LOOP study was lower than originally anticipated, leaving the study underpowered to investigate, whether OAC reduced the thromboembolic risk among DDAF patients. However, a non-significant trend towards thromboembolic risk reduction was found in the ICM group compared to standard care (HR 0.80 [CI 0.61–1.05]) [7]. This trend might partly be explained by the higher rate of OAC initiation in the ICM group.

The NOAH-AFNET 6 trial had a primary composite endpoint of cardiovascular death, stroke and/or systemic embolism. The control group received placebos, yet 53.9% of the patients were treated with aspirin at baseline. The study was underpowered due to premature termination at 21 months because preliminary results suggested futility for the efficacy of edoxaban. Nevertheless, a non-significant trend towards lower thromboembolic risk in the edoxaban group was identified, but it did not reach statistical significance (HR 0.65 [CI 0.39–1.07]). The absolute risk reduction (ARR) was 0.5% per patient-year, with a number needed to treat (NNT) of 200 patients to prevent one ischaemic stroke or systemic embolism. [25]. A later subgroup analysis found similar results in patients with DDAF duration > 24 h compared with < 24 h [31]. The same subgroup analysis concluded that patients with DDAF duration > 24 h were more likely to develop clinical AF than patients with duration < 24 h, 29.3% and 17.6%, respectively. This may have resulted in an underestimation of the efficacy of edoxaban because more patients with long-duration DDAF entered open-label treatment.

The primary endpoint of the ARTESiA trial was a composite of stroke and systemic embolism. The control group was treated with aspirin, which may have led to an underestimation of the thromboembolic risk reduction of apixaban. However, a significant risk reduction was identified in the apixaban arm compared with controls (HR 0.63 [CI 0.45–0.88]). The ARR was 0.46% per patient-year with an NNT of 217 patients to prevent one stroke or systemic embolism. Of note, apixaban significantly reduced the risk of severe stroke, as defined by the modified Rankin scale, with 19 severe strokes compared with 37 severe strokes in the aspirin group (HR 0.51 [CI 0.29–0.88]) [22]. A subsequent post-hoc study found that initiation of apixaban made no significant difference in stroke or systemic embolism in patients with a CHA_2_DS_2_-VASc score < 4 with 24 events in the apixaban group and 27 in the control group (HR 0.87 [CI 0.50–1.52]). In DDAF patients with a CHA_2_DS_2_-VASc score > 4, there were 18 and 38 events, respectively, (HR of 0.44 (CI 0.25–0.77) and an ARR of 3.95% in favour of apixaban. DDAF patients with a CHA_2_DS_2_-VASc score of 4 had a non-significant trend towards fewer strokes and systemic embolism with apixaban compared to aspirin (HR of 0.63 [CI 0.32–1.27]) [24].

In the meta-analysis of the ARTESiA and NOAH-AFNET 6 trials, DOAC treatment showed a significant risk reduction for ischaemic stroke with 67 (2%) among DOAC-treated patients and 98 (3%) in the control group (HR 0.68 [CI 0.5–0.92]). The ARR was 1% with an NNT of 100 patients to prevent one ischaemic stroke. There were 78 (2.4%) compared with 119 (3.6%) all-cause strokes or systemic embolisms in the DOAC and control group, respectively, leading to a significant risk reduction with DOAC treatment (HR 0.65 [CI 0.49–0.86]). The ARR was 1.2% with an NNT of 83 patients to prevent one stroke or systemic embolism [30].

A potential reason for the non-significant thromboembolic risk reduction in NOAH-AFNET 6 compared with ARTESiA was the lower number of events in NOAH-AFNET 6 resulting in the study being underpowered to detect a difference with DOAC treatment. The inclusion of cardiovascular death into the primary composite endpoint in NOAH-AFNET 6 might also have underestimated the effect of stroke risk reduction with edoxaban treatment since the included population rarely dies from stroke, but more often of old age and underlying cardiovascular disease [4,32].

A subgroup analysis of pooled data from ARTESiA and NOAH-AFNET 6 found a significantly reduced risk of stroke and systemic embolism in patients with vascular disease, with an annual rate in each study of 1.0% and 1.2%, respectively (IRR 0.55 (CI 0.38–0.78)), favouring DOAC treatment. The same effect was not identified in the subgroup without vascular disease [26].

## 6. Perspective

During the past decades, we have learned from pivotal trials in patients with clinical AF that treatment with warfarin or DOACs effectively reduces stroke and mortality risk [23,36,37,38,39,40]. Patients included in the early trials were at very high risk of stroke, with a calculated average stroke rate of 6.03% per year in the placebo/control groups and 3.8% per year in the aspirin groups, respectively, as reported in a meta-analysis by Hart and colleagues [23]. Comparable stroke rates were found in the aspirin group of the AVERROES trial (3.7% per year) [32].

In clinical AF, several prediction models exist to help evaluate the risk of stroke and the risk of bleeding before initiating treatment with OAC [41]. Current guidelines recommend withholding OAC in patients with an annual stroke risk < 1%, considering OAC treatment with a risk between 1–2%, and recommend OAC treatment with a risk > 2% [42,43]. Several guidelines recommend the use of the CHA_2_DS_2_-VASc-score, which is based on clinical parameters and is developed to identify persons with truly low risk of stroke defined as an annual stroke rate < 1% [17,44]. The ABC (Age, Biomarkers, and Clinical history)-stroke risk score is another validated prediction tool that differs from the CHA_2_DS_2_-VASc score by including cardiac biomarkers, i.e., plasma levels of NT-proBNP, and hs-TNT [45,46,47]. The ABC-stroke risk score has a significantly higher C-index for prediction of stroke and systemic embolism compared with the CHA_2_DS_2_-VASc score [48,49,50], but has not been validated in DDAF patients.

The recently published 2024 ESC guidelines for the management of AF recommend considering DOAC treatment in DDAF subgroups with a high estimated risk of stroke, who simultaneously have an absence of major bleeding risk factors, without giving clear recommendations on how to identify these patients. The ESC guidelines paper states that the DDAF burden/duration warranting OAC treatment remains unclear, but appropriate treatment and follow-up would be important regardless of the initial decision on OAC treatment [43]. The ACC/AHA/ACCP/HRS AF guidelines from 2023 give more concrete recommendations, yet are formulated carefully, emphasising shared decision-making, patient’s individual risk, and DDAF duration. OAC initiation in patients with DDAF duration > 24 h and a CHA_2_DS_2_-VASc-score ≥ 2 is given a class 2 recommendation. The same guideline recommends that in patients with a DDAF duration between 5 min and 24 h and a CHA_2_DS_2_-VASc-score ≥ 3, it may be reasonable to initiate OAC with a 2b recommendation (evidence level B-non-randomised studies) [42]. In the ACC/AHA/ACCP/HRS guidelines, the ASSERT study post-hoc analysis is highlighted as an argument for initiating OAC treatment in patients with DDAF duration > 24 h [29], which is supported by additional observational studies. In ARTESiA, patients with DDAF duration > 24 h were excluded, thus, not providing new evidence on long duration. In the NOAH-AFNET 6 trial, which saw no stroke risk reduction in patients receiving edoxaban, the median duration of DDAF episodes was 2.8 h [25]. This was only half of that (1.47 h) in the ARTESiA trial, which showed a statistically significant stroke risk reduction with apixaban [22]. A closer look at the data shows that 46.5% of the apixaban group also received aspirin, which may have had an effect on stroke prevention [26]. Additionally, in the LOOP study, the DDAF burden was low, despite a high prevalence of DDAF using an ICM [51]. An ARTESiA subgroup analysis found no association of baseline DDAF frequency or duration with thromboembolic risk [30], suggesting that a DDAF duration < 24 h is not an important factor in thromboembolic risk estimation in DDAF [30]. These results are in contrast to the TRENDS (relationship between daily atrial tachyarrhythmia burden from implantable device diagnostics and stroke risk) study in which patients with DDAF episodes with a median duration of ≥ 5.5 h had an increased stroke risk, yet 30% of the patients had already documented clinical AF at inclusion [5]. A subgroup analysis in NOAH-AFNET 6 found no difference in annual stroke rates associated with DDAF duration > 24 h, yet with only four strokes in this subgroup, it was underpowered [31]. An important finding in the same analysis was a strong association between long DDAF duration and the development of clinical AF leading to shorter median follow-up time due to censoring. In both ARTESiA and NOAH-AFNET 6, many patients developed clinical AF (Table 1). It is possible that patients with long DDAF duration could have a higher risk of stroke, not reflected by the two studies, due to a higher rate of progression to clinical AF (in ARTESiA also DDAF duration > 24 h), which led to censoring from analysis in both studies. Given the current conflicting results, a reassessment of the exact DDAF burden that should guide the initiation of OAC treatment is warranted. Therefore, future studies investigating the total DDAF burden and its progression over time are needed.

Although the predicted nominal stroke risk of the patients included in the ARTESiA and NOAH-AFNET 6 trials was high, with a mean or median CHA_2_DS_2_-VASc score of 4 corresponding to an annual stroke rate of 4.0% [22,25], the actual annual rate of ischaemic stroke in the control groups of these trials was surprisingly low (1.24% and 1.1% per year, respectively) and fits well with a CHA_2_DS_2_-VASc score of 1 [17]. A similar annual stroke rate was reported for the control group of the LOOP study (1.09% per year) [7], while patients included in the ASSERT trial had a slightly higher annual stroke rate of 1.69%, if DDAF was present within three months after enrolment but still below that corresponding to a CHA_2_DS_2_-VASc score of 2 [4].

The lower stroke rates in the recent studies compared with the older cohort studies might be at least partly explained by a general decline in the annual incidence of stroke over the last 30 years, which is likely due to better management of other stroke risk factors than AF, e.g., hypertension and hypercholesterolemia [52]. As such, a holistic approach to DDAF patients might be important, including assessment of whether other risk factors for stroke are well-controlled or if there is room for improvement, rather than predominantly focusing on OAC treatment in the individual patient. Another factor possibly contributing to the low annual stroke rate could be the large number of patients in both ARTESiA and NOAH-AFNET 6 (24% and 18.2%, respectively) entering open-label DOAC treatment due to developing clinical AF, which occurred more often in patients with longer DDAF duration, so that potential high risk-patients were excluded from the analysis.

With these factors in mind and considering that all patients in the control groups of ARTESiA, 53.9% in NOAH-AFNET 6, and 61% in the ASSERT trial received aspirin, which only modestly reduces stroke risk [23], this discrepancy between the anticipated and the actual stroke rates in patients with DDAF warrants further explanation. These patients do not seem to have the same stroke risk as patients with clinical AF, even if they have the same predicted nominal stroke risk, which raises the question of whether common stroke risk prediction schemes such as the CHA_2_DS_2_-VASc and ABC-stroke scores are applicable to patients with DDAF in their current form. Further research is warranted to elucidate how stroke risk prediction can be refined in patients with DDAF. Blood and imaging biomarkers such as NT-pro-BNP, troponins, markers of inflammation, haemostatic markers, and echocardiographic surrogate markers of atrial cardiomyopathy (e.g., left atrial diameter, volume, emptying fraction, or strain) could be options, but they have to prove their ability to better predict stroke in patients with DDAF. In light of the low stroke rate in this patient population, these studies will need a large number of patients and longer follow-up, making it challenging to conduct future studies. Another important question is whether the known clinical stroke risk factors in the CHA_2_DS_2_-VASc score have the same attributable risk in patients with DDAF as in those with clinical AF. The post-hoc analysis of ARTESiA concluded that DDAF patients with a CHA_2_DS_2_-VASc score > 4 had a 2.2% risk of stroke or systemic embolism per year, which corresponds to a CHA_2_DS_2_-VASc of 2 in clinical AF. This indicates that the CHA_2_DS_2_-VASc score might be applicable in DDAF patients, albeit the nominal risk score must be higher than in clinical AF. Future research is needed to shed more light on this.

When anticoagulating AF patients at lower risk of stroke, bleeding risk may begin outweighing the benefits of OAC treatment. Taking the results of the recent trials of NOAH-AFNET 6, ARTESiA, and LOOP together, we still do not know the “sweet spot” of the greatest benefit and the least harm of OAC treatment in patients with DDAF, i.e., which patients will benefit from initiating OAC treatment when AF is detected by an implantable device. Still, the question of whether to initiate OAC treatment in DDAF patients frequently arises in daily clinical practice. With the high NNT in both the ARTESiA and NOAH-AFNET 6 trials (Table 2) to avoid one thromboembolic event, it may be reasonable to use a more holistic approach in DDAF patients with a low to moderate thromboembolic risk, ensuring that other stroke risk factors are optimally treated as well. On the other hand, the NNH from major bleeding due to OAC treatment in DDAF patients were high in both ARTESiA and NOAH-AFNET 6 (Table 2), and in ARTESiA apixaban led to a significant reduction in severe strokes and was not associated with higher rates of fatal bleeding. This may be a strong argument for initiating OAC treatment in DDAF patients with high thromboembolic risk because stroke can lead to severe long-term disability [53]. Based on the subgroup analysis from the two randomised studies, patients who are likely to have a net benefit from OAC treatment are those with a CHA_2_DS_2_-VASc score > 4 and vascular disease due to a higher rate of thromboembolic events and at the same time less increased risk of major bleeding. Future studies with a clear definition of DDAF, assessment of burden and a sufficient number of participants with appropriate follow-up are needed. Stroke risk assessment may also be enriched by blood and imaging biomarkers that confer a hypercoagulable state and perhaps artificial intelligence (AI) might have the potential to optimise thromboembolic risk stratification. Furthermore, new anticoagulant drugs with a more favourable risk–benefit profile, such as factor XI/XIa inhibitors, might become relevant options in the future, if sufficient stroke risk reduction is proven. Moreover, it may not be sufficient to only look at the burden of DDAF, which must rather be seen together with different stages of atrial cardiomyopathy.

Until such studies have been conducted, we wish to emphasise the importance of shared decision-making and the obligation of clinicians to fully inform patients about the benefits and risks before considering OAC treatment in this patient population.

## Figures and Tables

**Table 1:** Patient characteristics and follow-up data in ARTESiA and NOAH-AFNET 6.

	NOAH-AFNET 6	ARTESiA
Number of patients	2536	4012
Intervention	Edoxaban	Apixaban
Comparator	Placebo/ASA	ASA
Age, years mean ± SD	77.5 ± 6.7	76.8 ± 7.6
Female sex (%)	37.4	36.1
BMI, kg/m^2^ ± SD	28.4 ± 4.8	28.8 ± 5.8
Ethnicity/race (%)	Not systemically reported (primarily Caucasian/European)	White European 94.2 Black African 2.2 Native Latin 0.5 South Asian 0.3 Native North American or Pacific Islander 0.3Other 2.4
CHA_2_DS_2_-VASc-score, median/mean (IQR/± SD)	4 (3–5)	3.9 ± 1.1
Diabetes mellitus (%)	25.9	29.1
Hypertension (%)	86.9	81.5
Heart failure (%)	27.4	28.3
Creatinine clearance, mL/min mean ± SD	66 ± 23.4	71.4 ± 28.7
Prior stroke, systemic embolism or TIA (%)	10	9
Vascular disease with indication ASA (%) *	56	45.9
ASA treatment at randomisation (%)	53.9	57.4
Number of patients received study drug (%)	1270 (50)	1989 (50)
ASA treatment in comparator group (%)	53.9	100
ASA treatment + intervention drug in intervention group (%)	NA	46.5
Device type:		
Pacemaker (%)	81.7	69.4
ICD (%)	7.4	13.8
CRT-ICD or CRT-pacemaker (%)	9.9	11.5
ICM (%)	1.0	5.2
Duration of DDAF episodes prior to enrolment, hours, median (IQR)	2.8 (0.8–9.4)	1.5 (0.2–5)
Follow-up, years, median/mean ± SD	1.8	3.5 ± 1.8
Development clinical AF (%) ^†^	18.2	24

Abbreviations: AF atrial fibrillation, ASA acetylsalicylic acid, BMI body mass index, CRT cardiac resynchronisation therapy, DDAF device-detected atrial fibrillation, ICD implantable cardioverter–defibrillator, ICM implantable cardiac monitor, IQR inter quartile range, NA not allowed, SD standard deviation, TIA transient ischaemic attack. * Unstable angina, prior myocardial infarction, prior coronary artery bypass grafting or percutaneous coronary intervention, prior transient ischaemic attack, prior stroke or established arterial disease. ^†^ ARTESiA definition includes detection with surface ECG and/or device-detected atrial fibrillation >24 h. NOAH-AFNET 6 includes detection by surface ECG.
